# Effects of artificially increased activation of the gluteus medius on ipsilateral lower limb muscles force during gait

**DOI:** 10.1371/journal.pone.0353269

**Published:** 2026-07-17

**Authors:** Omer Kursad Katirci, Diana Toderita, Anthony M. J. Bull

**Affiliations:** 1 Department of Bioengineering, Imperial College London, London, United Kingdom; 2 Department of Health Sciences, Afyonkarahisar Health Sciences University, Afyonkarahisar, Türkiye; University of Illinois Urbana-Champaign, UNITED STATES OF AMERICA

## Abstract

**Background:**

Functional Electrical Stimulation is widely used in rehabilitation to improve muscle function in individuals with neurological impairments. The gluteus medius muscle plays a key role in pelvic stability and balance during gait. The purpose of this study was to simulate functional electrical stimulation using musculoskeletal modelling to examine the effects of artificially increased gluteus medius activation on the activation of other ipsilateral lower limb muscles during the stance phase of gait in healthy adults.

**Methods:**

Musculoskeletal modelling simulations were conducted on gait data from 30 healthy participants to compare normal gait and gait modified by artificially increased gluteus medius activation. A static optimisation model was used to promote gluteus medius activation, replicating a functional electrical stimulation intervention. Changes in peak muscle forces, body-weight–normalised muscle impulse, force traces, peak hip joint reaction force, and hip joint reaction force traces were analysed.

**Results:**

Artificially enhanced gluteus medius activation significantly (p < 0.05) affected the peak force of seven muscles, the body-weight–normalised muscle impulse of eight muscles, peak hip joint reaction force, the force traces of ten muscles, and hip joint reaction force traces during stance.

**Conclusions:**

Increased gluteus medius muscle activation altered lower extremity muscle coordination through agonist–antagonist interactions, resulting in a redistribution of muscle forces that may be mechanistically associated with improved frontal-plane pelvic control and dynamic balance during walking. These findings, obtained in healthy individuals within an inverse dynamics-based musculoskeletal modelling framework, provide a mechanistic insight into how the increased contribution of the gluteus medius affects distal muscle loading patterns. Although this approach does not directly represent the phase-dependent or adaptive effects of functional electrical stimulation, the observed force distribution patterns may contribute to hypothesis generation for future studies investigating gait disorders.

## Background

Stroke causes disability and death. It is the most significant cause of long-term disability globally [1]. Hemiparesis is one of the most common symptoms in individuals who have had stroke, and it is characterised by weakness of the muscles on one side of the body [2]. After stroke, individuals with hemiparesis usually avoid putting weight on their paretic (affected) lower extremity [3]. This can be associated with the phenomenon of “learned non-use” [4]. The inability to bear weight on the paretic limb leads to substantial impairments in gait and balance. In these individuals, significant deficits in gait patterns, such as decreased paretic leg muscle activity and shortened step length, can be observed [5]. These deficiencies in weight-bearing ability impair balance during both standing and walking, leading to functional limitations.

A significant cause of asymmetric weight-bearing and transfer in individuals after stroke is the lack of activation in the hip abductor muscles during walking [6,7]. This deficiency specifically affects the pelvic stabilisation and hip joint alignment during the stance phase (approximately the first 60% of the gait cycle) of gait, which is a critical component of independent walking [8]. Individuals who cannot weight-bear on the paretic leg transfer their body weight (BW) more to the other leg, which causes gait asymmetries and increased energy expenditure [9]. Forced weight bearing on the paretic side may help correct these imbalances and increase overall muscle activity, as well as improve walking speed and functional mobility [10,11]. Therefore, an essential goal of post-stroke rehabilitation is to enable individuals to place more weight on their paretic extremities. Strengthening weight-bearing on the paretic side not only improves individuals’ walking, standing and balance performance but also improves their quality of life by increasing social participation [12].

Pelvic correction has positive effects on gait and balance in hemiplegic individuals. Studies show that targeted corrective external force applied to the pelvis increases activation of paretic side leg muscles and helps individuals transfer more weight to the paretic side [5,10,13]. External correction applied laterally to the coronal axis of the pelvis via a robotic device can improve paretic side muscle activations, weight transfer and symmetry in stroke patients [10]. This force may enhance muscle activity and improve gait symmetry by promoting forced use on the paretic side [10]. Furthermore, with this corrective force, an improvement in stride length asymmetry and an increase in weight-bearing ability on the paretic side were observed, which improves overall balance and gait speed by reducing energy expenditure [5,13]. A significant limitation of these studies is that the experiments were usually performed on a treadmill. This makes it difficult to fully evaluate the effectiveness in real-world walking conditions [14]. Furthermore, pelvic correction was achieved with the help of robotic devices in these studies, which are not yet widely available for clinical use due to their high cost.

The effects of applying a lateral force to the pelvis in the coronal axis with robotic devices can potentially be achieved by increasing the activation of the hip abductor and pelvic stabilisation muscles. Functional electrical stimulation (FES) is a means to increase the activation of these muscles. FES is currently used in many clinical conditions, including addressing foot drop syndrome after stroke. Therefore, using FES can potentially provide pelvic correction like that of robotic devices and make this more accessible and portable.

The gluteus medius (GM) provides 60% of the transverse cross-sectional area of all hip abductors [15,16]. In addition to the large cross-sectional area, the moment arm of the GM is higher than the other muscles that provide postural control in the medial-lateral direction [16,17]. Therefore, among hip abductors, the GM is most prominent in maintaining pelvic stability in the frontal plane [18]. Furthermore, its anatomical features, such as its relatively large size and superficial location, facilitate effective electrode placement and efficient stimulation compared to other hip abductors. These factors make the GM an optimal choice for studies targeting hip abductor function and its stabilisation role in gait.

Inverse dynamics-based musculoskeletal modelling (MSKm) is a mathematical method used to understand the dynamic interactions between anatomical structures such as bones, joints and muscles in the human body [19]. MSKm calculates the forces produced by muscles and contact loads on joints during a movement. Muscles are considered as force-generating structures, and equations of motion are solved using kinematic data, such as joint angles, and kinetic data, such as ground reaction forces; this is achieved through inverse dynamic analysis. The main challenge of the model is that the system is indeterminate, with infinite possible combinations of muscle activations able to solve the equations of motion [20]. Therefore, optimisation techniques are applied to solve the indeterminacy by minimising a physiologically relevant objective function, such as the sum of muscle stresses to a power which models repetitive activities such as walking and transfer activities [21]. This maximises muscular endurance by allowing larger muscles to produce more force to achieve the motion. Furthermore, the objective function can be artificially manipulated to increase the activation of specific muscles, which enables the effect of interventions to increase those activations, such as FES, to be analysed [20,22]. FES aims to improve motor function by activating specific muscles with electrical impulses. Adjusting the objective function enables MSKm to simulate the effects of FES on muscle activation in a non-invasive way and analyse using inverse dynamics how this results in changes to other muscle activations and other biomechanical variables.

It is well established from prior research that over-activating one muscle can alter the balance of muscle forces in other parts of the limb [20]. Building on this understanding, the aim of the present study was to quantify the changes in muscle forces across the lower limb due to overactivation of the hip abductors during the stance phase of level walking in healthy participants, as might be achieved with FES. The findings from this study in healthy individuals are expected to inform strategies for studying enhancing paretic side muscle activation in stroke rehabilitation.

## Methods

### Participants

Biomechanical data were obtained from the electronic records of an asymptomatic subgroup of participants of a prior cross-sectional study on the risk of osteoarthritis [23], which received ethical approval from the South West London Research Ethics Committee. Written informed consent was provided by all participants [23]. The data were accessed for secondary analysis in May 2023. The authors did not have access to any information that could identify individual participants during or after data analysis. From this dataset, a diverse group of 30 healthy participants (15 males, 15 females, age: 51 ± 18.8 years; height: 170.9 ± 10.4 cm; weight: 72.9 ± 11.8 kg) were identified to create a varied participant population. To ensure the study’s findings are applicable to a broad population, participants aged 18–90 years were included, reflecting the age distribution commonly observed in stroke populations.

The inclusion criteria and other details for this study were thoroughly detailed in a previous study [23]; in summary, participants with neurological, rheumatoid or other systemic inflammatory arthritis, a body mass index of > 35 kg/m^2^ or had undergone previous surgical treatment for knee OA were excluded from the study.

### Experimental protocol

Detailed information regarding the experimental protocol has been provided previously (23). In summary, a 10-camera Vicon motion capture system (T160, Vicon Motion System Ltd., Oxford, UK −100 Hz) and two portable force plates (Kistler Type 9286B, Kistler Instrumente AG, Winterthur, Switzerland – 1000 Hz) were used to collect gait data while participants walked on a 6m walkway at their self-selected speed. Twenty-three retro-reflective markers were placed on the participants’ thorax, pelvis, and lower extremities [24] was used for this experiment; twenty-three retro-reflective markers were placed on the participants’ thorax, pelvis, and lower extremities. Four clusters, each containing three markers, were positioned on the left and right thigh and calf segments to define joint centres and anatomical frames. For each participant, single static and five dynamic trials were recorded [23].

### Modelling and data analysis

Gait cycle events were labelled using Vicon Nexus (VICON 2.15.0, Oxford Metrics Group, UK) software based on a 30 N force threshold [25]; missing data points were interpolated using this software. The resulting C3D files were then further processed in MATLAB (The Mathworks Inc., Natwick, MA, USA). A fourth-order Butterworth filter with a cut-off frequency of 6 Hz [26–28], with no phase lag, was employed to filter the marker locations and ground reaction forces before the MSKm input. Kinematic and kinetic outputs were time-normalised to 100% of the gait cycle (heel strike to consecutive ipsilateral heel strike) but focused only on the stance phase (approximately first 60% of the gait cycle) to mimic FES devices with a footswitch. An open-source MSKm software, Freebody (v2.2), was used to carry out inverse kinematics, inverse dynamics and MSKm simulations [29,30]. The anatomical geometry for participants was determined according to the linear scaling method, in which the closest datasets were chosen based on similar mass, gender and limb length to pelvis width ratio [22].

All muscle force and joint reaction force (JRF) data were normalised to subject-specific BW, and analyses were performed on these values. Determining a universal threshold for clinically significant changes in muscle force during walking is inherently complex, as individual biomechanics, muscle function, joint structure and phase-specific gait goals all need to be considered. In our study, we adopted a 10% BW threshold to identify substantial changes in muscle force output and focused on muscles exhibiting ≥10% BW changes (on an individual level). While this exact threshold is not universally established in the literature, it is informed by previous findings suggesting that force perturbations approaching 10% BW can produce measurable effects on gait kinematics and neuromuscular control [10].

Muscle forces are key contributors to joint torque generation and maintaining neuromuscular control during walking. Therefore, we considered a 10% BW change in an individual muscle as a practical and biomechanically grounded cutoff that balances sensitivity to physiologically meaningful adaptations with the need to exclude trivial variations unlikely to reflect functional significance. This threshold facilitates the interpretation of intervention effects in a consistent and interpretable manner.

The existing literature provides both EMG-based and mechanical-outcome validation supporting the use of the FreeBody musculoskeletal model to study the effects of FES during gait [20,22,30,31]. In this study, the approach of modifying the objective function associated with the intended muscle was employed, aiming to promote its activation and mimic the effects of the FES intervention. Using the collected gait data, an optimisation process was carried out considering both the original (1) and modified (2) objective functions to evaluate the effects of enhanced muscle activation without changing the kinematics. For each trial, two distinct optimisation procedures were driven: one to simulate normal gait as a control and the other to simulate gait with augmented activation of the GM. The objective functions used are detailed as follows [20]:


$$\begin{array}{c} \text{Minimise J}=\;\sum_{i=\text{1}}^{n}{\left(\frac{F_{i}}{F_{imax}}\right)}^{\text{3}} \end{array}$$ (1)

$$\begin{array}{c} \text{Minimise J }=\;\sum_{i=\text{1}}^{n}c.{\left(\frac{F_{i}}{F_{imax}}\right)}^{\text{3}} \end{array}$$ (2)

$$\begin{array}{c} \;=\left\{ \begin{array}{c} \;\text{0}.\text{1}\text{0}\;for\;GM\;\\ \text{1}\;for\;all\;other\;muscles \end{array}\right. \end{array}$$

**J** *= sum of cubed muscle activations,* $F_{i}$*= the force of element* ***i****,* $F_{imax}$*= maximum force potential of element* ***i****,* ***n*** *is the total number of muscle elements (163),* **c** *is a coefficient, the lower it is, then the higher will be the activation of the specified muscle.*

For each muscle element, the maximum potential force (*Fimax*) was obtained as the product of its physiological cross-sectional area [32] and a constant maximum muscle stress value (60 N/cm^2^) [33]. Introducing a variable weighting factor ‘c’ allows the model to adjust how individual muscles contribute to the total force production defined by the input kinematic and kinetic data. Muscles assigned lower weighting values are prioritised within the optimisation process, as increases in their activation have a smaller impact on the cost function. As a result, these muscles exhibit greater simulated activation compared with others. When (1) and (2) are compared under identical kinematic and kinetic conditions, the latter formulation yields higher predicted activation for the GM. To satisfy the model’s equations of motion, this change is accompanied by compensatory adjustments in the forces produced by other muscles and in the JRFs. To quantify how increased GM activation influences the force distribution among other lower-limb muscles, the simulated forces of 37 muscles and hip JRF were examined. The coefficient (c) in the optimisation equation for the stimulated muscle was set to 0.10 [20,22,34]. This coefficient is not a physiological constant but a weighting factor that can be adjusted according to the model’s targeted stimulation effect. In addition, it has been reported that similar values (c = 0.10–0.20) are used in the literature to represent low–medium level FES conditions and that the model outputs are validated with EMG data [22].

### Statistical methods

SPSS 29 (IBM SPSS Statistics 29.0, IBM Corp., USA) was utilised to analyse the data from normal and activated conditions. Significance was set at p < 0.05.

Normality was tested using the Shapiro-Wilk test, and Q-Q plots were visually analysed. It was found that some muscles’ peak and the BW–normalised muscle impulse were normally distributed while others were not. When the data were normally distributed, a Paired Sample t-test was applied, and when they were not, the Wilcoxon test was applied.

Statistical Parametric Mapping (SPM) [35–37] was used to compare the stance phase time series to understand how the sub-phases of gait change between normal and activated conditions. Depending on the data distribution, parametric or non-parametric two-tailed paired sample t-tests were performed to determine the biomechanical differences between these two conditions. Data were analysed in terms of peak hip JRF and its traces over staff, peak muscle force and BW–normalised muscle force impulse. BW–normalised muscle force impulse during the stance phase was calculated by integrating muscle force over time to provide information regarding mechanical loading during the stance phase. Force trajectories were time-normalised according to the gait cycle, and the impulse value was obtained by summing the force values obtained throughout the stance phase (subject-specific, calculated as a percentage) and multiplied by the participant-specific time step (Δt).

## Results

**Fig 1:**
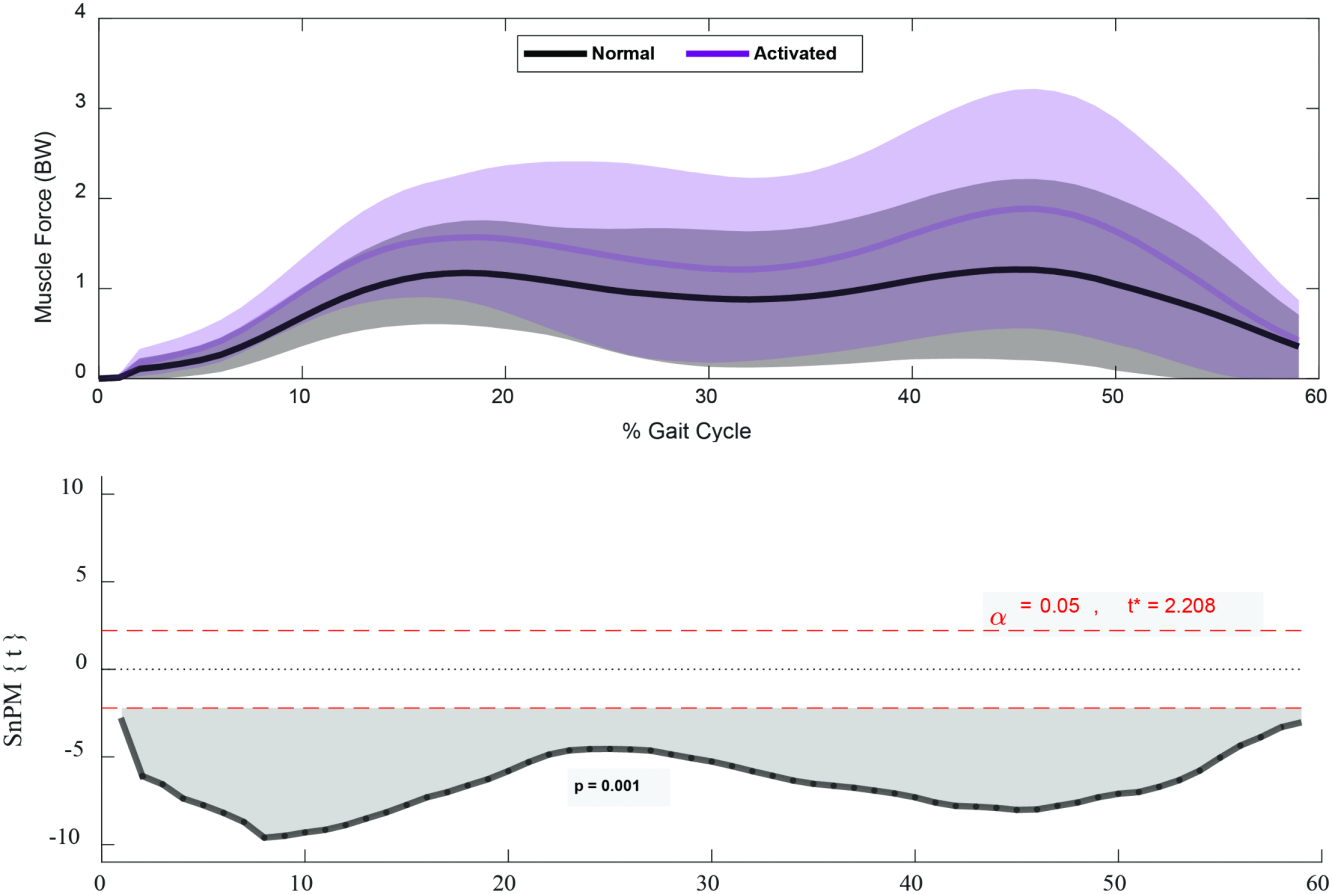
Time-resolved comparison of GM muscle forces between normal and augmented gait. Top: GM force changes during the stance phase of gait. Bottom: Statistical parametric mapping result, with the grey box highlighting the statistical differences. t* = The critical threshold of the paired sample t-test statistic SPM {t}, α = Significance level.

(Fig 1) shows the change in force generation of a normal GM muscle and a model-activated GM muscle during the stance phase of a walking cycle. The peak force of the manipulated GM muscle increased by 0.66 times the BW of the normal GM muscle. There were significant differences between the two conditions in the peak force (p < 0.001), the BW–normalised muscle force impulse (p < 0.001) and the force graphs throughout the movement (when looking with SPM; p < 0.001).

GM activation led to force alterations in all other muscles. A clinically important change in peak muscle force was set at 10% BW, and these results only are presented. These are: adductor brevis (ADB; p < 0.001), adductor longus (ADL; p = 0.688), GMIN (p < 0.001), gastrocnemius (GSTR; p = 0.107), psoas major (PM; p < 0.001), rectus femoris (RF; p = 0.043), semimembranosus (SMB; p = 0.009), soleus (SOL; p < 0.001), vastus lateralis (VL; p < 0.001), and vastus medialis (VM; p < 0.001) (Table 1).

**Table 1:** Changes in the peak forces (BW) under normal and activated conditions of GM during the stance phase of gait among all muscles with peak≥0.1 BW differences.

Muscles	Condition	Median *or* Mean (BW)	Range (min-max) *or* SD	Z-Value *or* t-Value	p-Value
**ADB** ^**†**^	Normal	0.1472	0.02-0.54	−4.78	0.000^**^
	Activated	0.2095	0.07-0.92		
**ADL** ^**†**^	Normal	0.5140	0.15-1.39	−0.40	0.688
	Activated	0.4925	0.15-1.92		
**GMIN** ^**†**^	Normal	0.3008	0.07-1.30	−4.70	0.000^**^
	Activated	0.2722	0.06-0.84		
**GSTR**	Normal	2.7938	1.1792	1.66	0.107
	Activated	2.6564	1.0784		
**PM** ^**†**^	Normal	0.7497	0.29-2.71	−4.57	0.000^**^
	Activated	0.6153	0.26-2.11		
**RF**	Normal	2.6188	1.1698	2.12	0.043^*^
	Activated	2.4999	1.0170		
**SOL** ^**†**^	Normal	0.9184	0.00-7.79	−3.77	0.000^**^
	Activated	1.1258	0.00-7.79		
**SMB**	Normal	0.6497	0.3091	2.79	0.009^*^
	Activated	0.5369	0.2730		
**VL** ^**†**^	Normal	0.3437	0.00-2.23	−4.00	0.000^**^
	Activated	0.4627	0.00-1.85		
**VM**	Normal	0.7327	0.3542	4.94	0.000^**^
	Activated	0.5453	0.2131		

Note: The t-value represents the size and direction of the mean difference between the paired groups. The Z-value represents the standardised test statistic for the Wilcoxon signed-rank test. (SD: standard deviation, ^**†**^**:** non-normally distributed data, * = p < 0.05, ** = p < 0.001).

Significant changes were observed in the BW–normalised muscle force impulse for nine muscles above the peak 10% BW threshold (ADB, p < 0.001; ADL, p < 0.001; GSTR, p < 0.001; GMIN, p < 0.001; SMB, p < 0.001; SOL, p < 0.001; RF, p < 0.001; VM, p < 0.001; Table 2).

**Table 2:** Changes in the BW–normalised muscle force impulse (BW.s) graphs under normal and activated conditions during the stance phase of gait among all muscles with peak≥ 10% BW differences.

Muscles	Condition	Median *or* Mean (BW.s)	Range (min-max) *or* SD	Z-Value *or* t-Value	p-Value
**ADB** ^**†**^	Normal	0.0146	0.0005-0.1262	−4.782	0.000^**^
	Activated	0.0341	0.0063-0.256		
**ADL** ^**†**^	Normal	0.0618	0.008-0.3809	−3.980	0.000^**^
	Activated	0.0796	0.008-0.5542		
**GSTR** ^**†**^	Normal	0.6653	0.013-1.783	−4.083	0.000^**^
	Activated	0.6023	0.0111-1.7289		
**GMIN** ^**†**^	Normal	0.0869	0.0139-0.4681	−4.762	0.000^**^
	Activated	0.0617	0.0135-0.3087		
**PM** ^**†**^	Normal	0.1276	0.0436-0.8928	−0.689	0.491
	Activated	0.1305	0.0437-0.7434		
**RF**	Normal	0.6531	0.325	3.926	0.000^**^
	Activated	0.5947	0.299		
**SMB** ^**†**^	Normal	0.096	0.00-0.3149	−4.021	0.000^**^
	Activated	0.0569	0.00-0.2632		
**SOL** ^**†**^	Normal	0.2775	0.00-2.1377	−4.042	0.000^**^
	Activated	0.3497	0.00-2.136		
**VL** ^**†**^	Normal	0.0066	0.00-0.6885	−1.759	0.079
	Activated	0.0277	0.00-0.4617		
**VM**	Normal	0.1258	0.0704	5.232	0.000^**^
	Activated	0.0879	0.0402		

Note: The t-value represents the size and direction of the mean difference between the paired groups. The Z-value represents the standardised test statistic for the Wilcoxon signed-rank test. (SD: standard deviation, ^**†**^**:** non-normally distributed data, * = p < 0.05, ** = p < 0.001).

**Fig 2:**
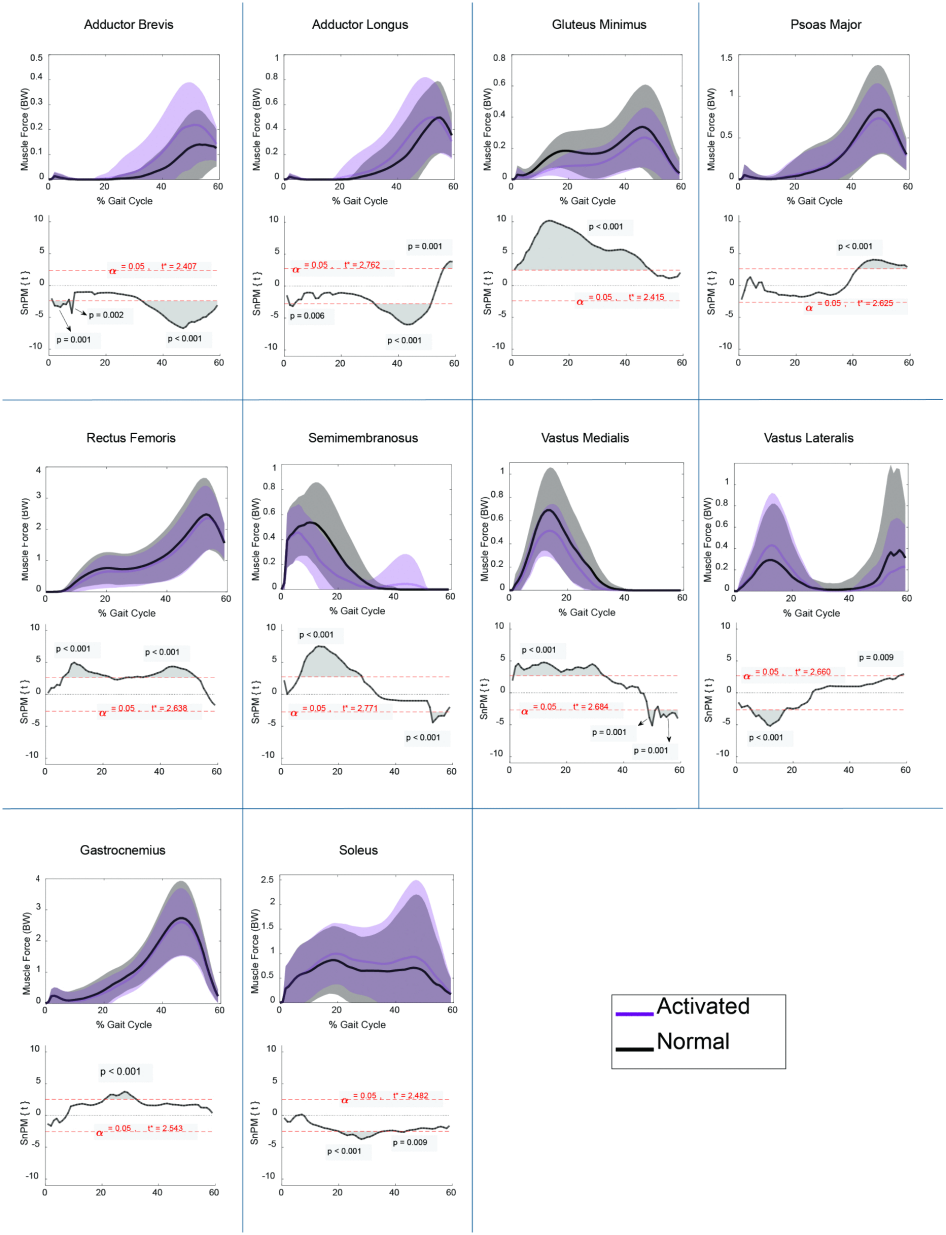
Time-resolved comparison of muscle forces between normal and augmented GM gait conditions. Top: Muscle forces in two conditions (normal and augmented gait) during the stance phase. Bottom: Statistical parametric mapping results, with grey boxes highlighting statistical differences. t* = The critical threshold of the paired sample t-test statistic SPM {t}, α = Significance level.

The SPM analysis of muscle forces during the stance phase of the gait cycle found (Fig 2):

- increased muscle force of ADB in the initial contact, terminal stance and preswing phases for the activated condition (p < 0.001);
- increased force of ADL in the initial contact (p = 0.006), terminal stance (p < 0.001) phases and decreased force in the preswing phase (p < 0.001);
- lower GSTR force in the midstance phase in the activated condition (p < 0.001);
- lower GMIN force during the whole stance phase except for the preswing phase (p < 0.001);
- lower PM force from the midstance phase (p < 0.001);
- lower RF force in the loading response phase and terminal stance (p < 0.001);
- lower SMB force in the loading response and midstance phases and increased in the preswing phase (p < 0.001)
- increased SOL force between the midstance phase and the middle of the terminal stance phase (p < 0.001);
- higher VL force in the loading response phase compared to the normal condition (p < 0.001); and it decreased in the preswing phase (p = 0.009); and
- decreased VM force at the beginning of the gait cycle until the terminal stance phase (0.001).

**Fig 3:**
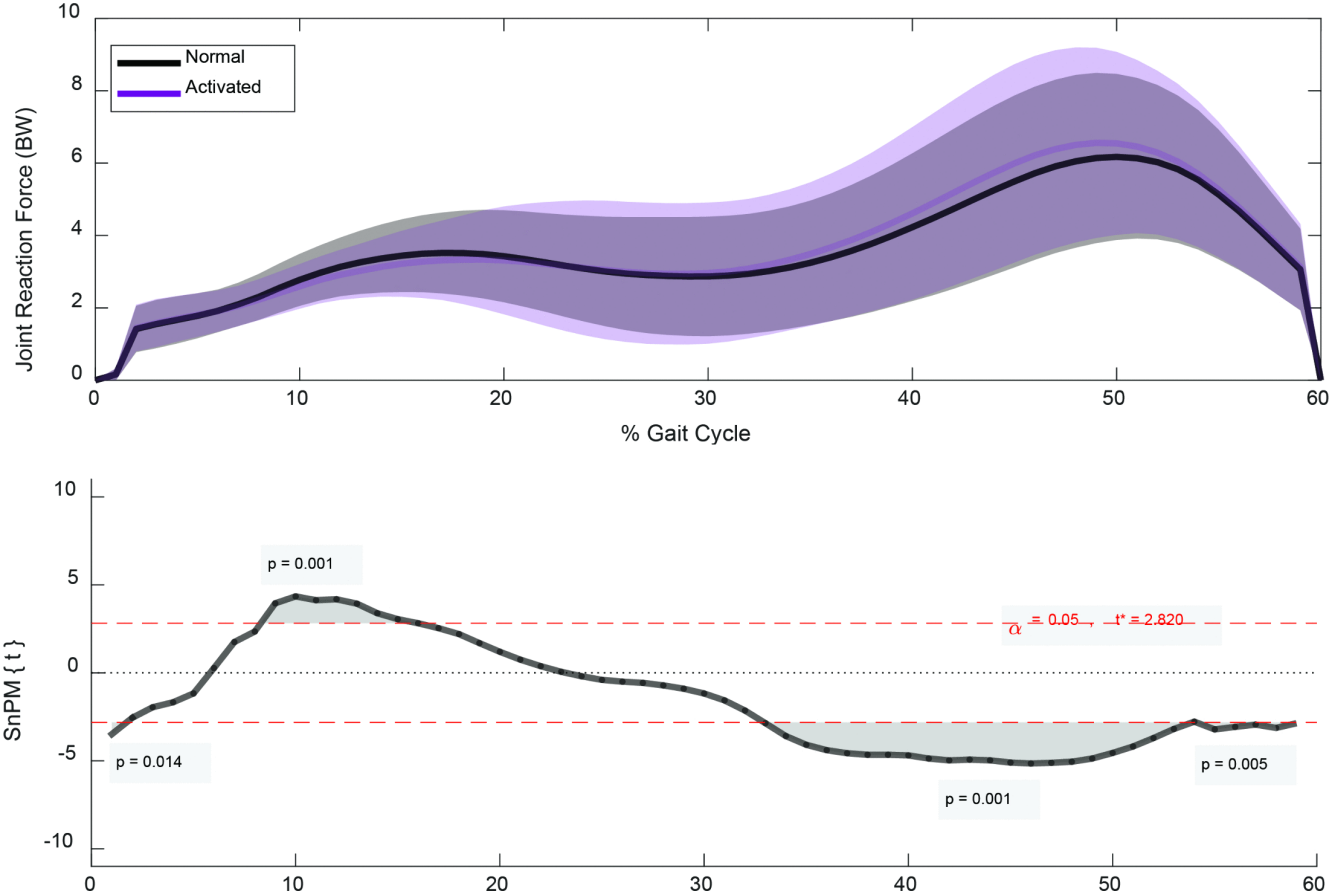
Time-resolved comparison of Hip JRF between normal and activated GM gait conditions. Top: Mean (±SD) hip JRF in two conditions (normal and activated gait) during the stance phase. Bottom: Statistical parametric mapping results, with grey boxes highlighting statistical differences. t* = The critical threshold of SPM {t}, α = Significance level.

Peak hip JRF increased significantly under the activated condition (p < 0.001, Table 3) and there was a significant increase in JRF in the activated condition during the early stance phase of gait (p = 0.001, Fig 3). In contrast, a significantly reduced JRF was observed in the activated condition during mid-to-late stance (p = 0.001, p = 0.005; Fig 3).

**Table 3:** Peak hip joint reaction forces (BW) under normal and activated conditions of GM during the stance phase of gait.

	Condition	Median	Range (min-max)	Z-Value	p-Value
**Hip JRF Peak** ^**†**^	Normal	6.01	3.65-15.91	−3.548	0.000^**^
	Activated	6.37	3.83-18.46		

Note: The Z-value represents the standardised test statistic for the Wilcoxon signed-rank test. (^**†**^**:** non-normally distributed data, ** = p < 0.001).

## Discussion

In this study, we demonstrated the effects of artificially increased hip abductor muscle activation on the forces of other ipsilateral lower extremity muscles during the stance phase of gait in healthy adults, simulating FES. MSKm showed that increased GM activation significantly altered the peak force of seven muscles, the BW–normalised muscle force impulse of eight muscles, the force of ten muscles and the hip JRF during different subphases of stance. We believe the main reason for these changes lies in the agonist-antagonist relationships between these muscles where the increased activation of the GM would require both increased and decreased activations of other muscles. This is expected as previous studies have reported that changes in the activation of one muscle can influence the activation patterns of other muscles, especially within functional groups characterised by agonist-antagonist relationships [38–40].

The activation of the hip abductors plays a critical role in triggering a chain of agonist-antagonist relationships among lower limb muscles. This relationship is crucial for maintaining dynamic balance, ensuring efficient force production, and providing stability during complex movements like walking, [38–40] particularly around the knee and ankle joints [40].

Hip abductors, particularly the GM, not only provide pelvic stabilisation in the frontal plane but also make a considerable contribution to carrying BW during the stance phase. The literature indicates that abductor capacity contributes to balance and mobility and has been quantitatively measured at the muscle level [41,42]. Recent studies have shown that stronger or more effectively recruited hip abductors are associated with better balance performance and walking function, highlighting their systemic importance in locomotion [42,43]. Furthermore, in gait, frontal plane control, mediolateral step placement, and active modulation of ground reaction forces are required; in this context, the GM exhibits task-dependent activation for stabilising the pelvis on the stance side [43,44]. In post-stroke gait, pelvic restraint/assistance applications that necessitate loading on the paretic side increase paretic side abductor EMG activity and improve pelvic displacement symmetry; this is consistent with the “forced use” mechanism [10,45]. These data indicate that the findings observed in our simulation—increased GM activation enhancing pelvic stability and reshaping the force distribution between hip muscles—are consistent with the abductors’ dual support and stabilisation functions. Specifically, increased GM activation significantly increased antagonist ADB force during terminal stance and pre-swing, and ADL force during terminal stance, while decreasing agonist GMIN force throughout the stance phase. This indicates that strengthening the abductors alters the force balance within the same limb in a manner consistent with their broader functional role.

When GM activation increases, its contribution to pelvic stabilisation increases, thereby reducing the contribution of the agonist PM and the biarticular RF muscle to pelvic stabilisation and subsequently reducing the activation of these muscles. Simultaneously, VL force increased during the loading response phase but decreased during the pre-swing phase. In contrast, VM force decreased from the beginning of the gait cycle until the terminal stance phase. These phase-dependent fluctuations reflect compensatory adjustments for the reduced RF force, helping to maintain overall knee extensor balance. Although VL and VM are usually co-activated in human gait, in our model, their force diverged slightly across phases (e.g., VL increased while VM decreased in early stance). We interpret this not as independent neural control but as an artefact of optimisation-based redistribution of force among synergistic knee extensors. Likewise, the reduction in knee extensor muscle activation results in decreased activation of the antagonist knee flexor, the SMB. Finally, the activation of the GSTR muscle, which crosses two joints, decreases in response to the reduced activation of knee extensors, as it serves as an antagonist. We estimate that this reduction in GSTR activation is compensated for by increased activation of the SOL muscle.

Pelvic correction interventions are critical in improving gait function in stroke patients. These interventions are typically applied to stabilise the pelvis during walking, correct movement asymmetry by increasing the load on the paretic lower limb and enhance muscle activation on the paretic side. Targeted forces explicitly applied to the pelvis have been shown to both increase muscle activity in the paretic lower limb and improve overall gait symmetry [13]. Studies have demonstrated that corrective forces applied to the pelvis during walking in stroke patients activate the ‘forced use’ principle by increasing the load on the paretic lower limb, thereby enhancing the activation of muscles such as the medial hamstring, tibialis anterior (TA), SOL, and GM [10,13]. In addition to increasing muscle activation in the paretic lower limb, these forces prolong the load-bearing time on the paretic side, promoting motor learning and improving movement efficiency [5,13]. We propose that a similar effect could potentially be achieved by increasing hip abductor muscle activation with FES, though this hypothesis requires direct testing in stroke survivors. In our model, based on healthy participant data, increasing GM activation improved frontal-plane pelvic stability and altered within-limb muscle balance; however, because kinematics were constrained and only GM was up-weighted, these changes did not substantially propagate to the TA, while SOL force showed a significant increase between mid-stance and the middle of terminal stance. This increase in SOL force may reflect a compensatory mechanism to maintain ankle joint force balance during stance, though whether such compensatory patterns would manifest similarly in individuals with hemiparesis remains to be established.

It has been hypothesised that FES applied to the GM muscle may be a promising intervention for improving walking function in stroke patients. Since this muscle plays a fundamental role in providing pelvic stability and preventing pelvic drop, FES application can lead to significant improvements in gait for stroke patients [46]. However, existing literature has often focused on the combined application of FES to the GM and TA muscles in stroke patients, with limited attention paid to the effects of this intervention on weight-bearing and paretic-side muscle activation. Notably, increasing the activation of these two muscles has shown significant benefits in improving both walking speed and balance [47,48].

In another study that applied FES to these two muscles, it was shown that this intervention contributed to a more normalised gait pattern, potentially reducing energy expenditure during walking and improving both static and dynamic balance [49]. A study by Araki et al. demonstrated that FES application to the GM and TA significantly increased walking speed by increasing stride length and expanding the range of motion. This study also showed that the combination of FES on the GM and TA muscles has a synergistic effect on walking performance, especially for individuals using assistive devices [48]. These findings are associated with both enhanced muscle activation and stability during walking. In our study, musculoskeletal simulations based on healthy participant data suggested that increased GM activation may induce changes in lower extremity muscle force distribution; however, whether these effects translate to stroke survivors remains an important question for future research. Although previous studies reported that combined GM and TA stimulation can synergistically improve gait performance [48,49], in our model TA activation did not increase despite higher GM activation. This discrepancy likely reflects the fact that only GM was up weighted in our cost function and gait kinematics were constrained. Synergistic effects may require simultaneous stimulation of both GM and TA under unconstrained kinematics using a forward dynamics simulation approach.

Previous studies have demonstrated that FES applied to the GM can alter knee joint loading patterns [20] and that targeted stimulation of muscles, such as the biceps femoris long head, may enhance knee joint stability [34]. In line with these findings, in our current study, increased GM activation led to a significant increase in the hip JRF peak. The increase in JRF observed during the early stance phase reflects increased joint compression forces resulting directly from FES-simulated muscle contraction, whilst the decreases during the mid- and late-stance phases suggest a more efficient load distribution following improved pelvic stabilisation. This biomechanical balance is consistently observed in studies examining targeted single-muscle FES interventions [20,34]. Our simulations demonstrated that increasing GM activation not only improved pelvic stabilisation but also redistributed forces in both agonist and antagonist muscles. These adaptations suggest that GM stimulation has system-wide effects on lower-limb muscle coordination. Although our model was constrained to fixed kinematics, the observed within-limb force redistributions may provide insight into mechanisms through which GM-targeted FES could support more stable and symmetrical gait in individuals post-stroke.

There are limitations to this study. Firstly, the standard objective function used in the study may not be directly adaptable to the gait patterns of stroke individuals. Stroke individuals have different muscle activation patterns and kinematic changes, which may limit the accuracy of the simulation results. However, prior work in healthy controls has shown that the muscular activation changes due to FES stimulation for non-stimulated muscles can be predicted accurately [22]. We acknowledge these limitations and the limited direct clinical relevance to the stroke population; however, this study provides valuable insights and cues for future experimental studies. Secondly, the study used an inverse dynamic MSKm, which does not account for kinematic changes. Potential changes in gait kinematics caused by FES application are ignored. This may mean that kinematic changes that may be encountered in the real world may not be fully reflected in the model. It is important to acknowledge that increased muscle activation induced by FES does not necessarily translate into direct functional improvements in movement every time. This could be addressed in future through forward dynamics simulations and gait experiments. In addition, in this application, the muscle contributions were adjusted throughout the gait cycle and not for specific portions of the cycle. This could be addressed by creating an adaptive objective function. Lastly, artificially increased activation of the GM muscle produced varying degrees of change in the activation of 37 other ipsilateral lower extremity muscles. However, most of these changes were minimal and unlikely to hold clinical significance. We used a 10% BW threshold to identify functionally meaningful changes in activation; however, this is not a universal threshold and is potentially arbitrary. Alternative approaches could be to have a threshold that is dynamic and scaled to the maximum muscle activation.

## Conclusion

This modelling study using healthy participant data demonstrates that artificially increasing gluteus medius activation produces significant effects on the activation patterns of other lower extremity muscles during the stance phase of walking. These effects are associated with the redistribution of muscle force arising through agonist–antagonist interactions, contributing to stability and force sharing during walking. These findings in healthy individuals provide a mechanistic framework for how changes in muscle activation may affect gait mechanics in the context of pelvic instability, which is frequently observed after stroke. Therefore, approaches aimed at increasing gluteus medius activation may be considered a potential target for interventions supporting pelvic stabilisation in stroke individuals; however, the clinical validity of this potential must be confirmed by further experimental and clinical studies.

## References

[1] GBD 2019 Stroke Collaborators. Global, regional, and national burden of stroke and its risk factors, 1990-2019: a systematic analysis for the Global Burden of Disease Study 2019. Lancet Neurol. 2021;20(10):795–820. doi: 10.1016/S1474-4422(21)00252-0 34487721 PMC8443449

[2] Hilkens NA, Casolla B, Leung TW, de Leeuw FE. Stroke. Lancet. 2024;403:2820–36. doi: 10.1016/S0140-6736(24)00642-1 38759664

[3] Hendrickson J, Patterson KK, Inness EL, McIlroy WE, Mansfield A. Relationship between asymmetry of quiet standing balance control and walking post-stroke. Gait Posture. 2014;39(1):177–81. doi: 10.1016/j.gaitpost.2013.06.022 23877032

[4] Kwakkel G, Veerbeek JM, van Wegen EEH, Wolf SL. Constraint-induced movement therapy after stroke. Lancet Neurol. 2015;14(2):224–34. doi: 10.1016/S1474-4422(14)70160-7 25772900 PMC4361809

[5] Hsu C-J, Kim J, Roth EJ, Rymer WZ, Wu M. Use of pelvic corrective force with visual feedback improves paretic leg muscle activities and gait performance after stroke. IEEE Trans Neural Syst Rehabil Eng. 2019;27(12):2353–60. doi: 10.1109/TNSRE.2019.2950226 31675335 PMC6939618

[6] Lanza MB, Gray VL. The effects of stroke on weight transfer before voluntary lateral and forward steps. Front Neurol. 2022;13:891439. doi: 10.3389/fneur.2022.891439 35937060 PMC9355404

[7] Kirker SG, Simpson DS, Jenner JR, Wing AM. Stepping before standing: hip muscle function in stepping and standing balance after stroke. J Neurol Neurosurg Psychiatry. 2000;68(4):458–64. doi: 10.1136/jnnp.68.4.458 10727481 PMC1736885

[8] Pandy MG, Andriacchi TP. Muscle and joint function in human locomotion. Annu Rev Biomed Eng. 2010;12:401–33. doi: 10.1146/annurev-bioeng-070909-105259 20617942

[9] Awad LN, Palmer JA, Pohlig RT, Binder-Macleod SA, Reisman DS. Walking speed and step length asymmetry modify the energy cost of walking after stroke. Neurorehabil Neural Repair. 2015;29(5):416–23. doi: 10.1177/1545968314552528 25288581 PMC4385745

[10] Hsu C-J, Kim J, Tang R, Roth EJ, Rymer WZ, Wu M. Applying a pelvic corrective force induces forced use of the paretic leg and improves paretic leg EMG activities of individuals post-stroke during treadmill walking. Clin Neurophysiol. 2017;128(10):1915–22. doi: 10.1016/j.clinph.2017.07.409 28826022 PMC5593794

[11] Aruin AS, Rao N, Sharma A, Chaudhuri G. Compelled body weight shift approach in rehabilitation of individuals with chronic stroke. Top Stroke Rehabil. 2012;19(6):556–63. doi: 10.1310/tsr1906-556 23192720 PMC3676671

[12] Hsiao H-Y, Gray VL, Borrelli J, Rogers MW. Biomechanical control of paretic lower limb during imposed weight transfer in individuals post-stroke. J Neuroeng Rehabil. 2020;17(1):140. doi: 10.1186/s12984-020-00768-1 33109225 PMC7590464

[13] Park SH, Lin J-T, Dee W, Hsu C-J, Roth EJ, Rymer WZ, et al. Targeted pelvic constraint force induces enhanced use of the paretic leg during walking in persons post-stroke. IEEE Trans Neural Syst Rehabil Eng. 2020;28(10):2184–93. doi: 10.1109/TNSRE.2020.3018397 32816677 PMC7652375

[14] Vickery-Howe DM, Bonanno DR, Dascombe BJ, Drain JR, Clarke AC, Hoolihan B, et al. Physiological, perceptual, and biomechanical differences between treadmill and overground walking in healthy adults: a systematic review and meta-analysis. J Sports Sci. 2023;41(23):2088–120. doi: 10.1080/02640414.2024.2312481 38350022

[15] Winter DA, Prince F, Frank JS, Powell C, Zabjek KF. Unified theory regarding A/P and M/L balance in quiet stance. J Neurophysiol. 1996;75(6):2334–43. doi: 10.1152/jn.1996.75.6.2334 8793746

[16] Lee S-P, Souza RB, Powers CM. The influence of hip abductor muscle performance on dynamic postural stability in females with patellofemoral pain. Gait Posture. 2012;36(3):425–9. doi: 10.1016/j.gaitpost.2012.03.024 22607792

[17] Neumann DA. Kinesiology of the hip: a focus on muscular actions. J Orthop Sports Phys Ther. 2010;40(2):82–94. doi: 10.2519/jospt.2010.3025 20118525

[18] Porto JM, Freire Júnior RC, Bocarde L, Fernandes JA, Marques NR, Rodrigues NC, et al. Contribution of hip abductor-adductor muscles on static and dynamic balance of community-dwelling older adults. Aging Clin Exp Res. 2019;31(5):621–7. doi: 10.1007/s40520-018-1025-7 30182152

[19] Smith SHL, Coppack RJ, van den Bogert AJ, Bennett AN, Bull AMJ. Review of musculoskeletal modelling in a clinical setting: current use in rehabilitation design, surgical decision making and healthcare interventions. Clin Biomech (Bristol). 2021;83:105292. doi: 10.1016/j.clinbiomech.2021.105292 33588135

[20] Rane L, Bull AMJ. Functional electrical stimulation of gluteus medius reduces the medial joint reaction force of the knee during level walking. Arthritis Res Ther. 2016;18(1):255. doi: 10.1186/s13075-016-1155-2 27809923 PMC5094077

[21] Crowninshield RD, Brand RA. A physiologically based criterion of muscle force prediction in locomotion. J Biomech. 1981;14(11):793–801. doi: 10.1016/0021-9290(81)90035-x 7334039

[22] Ding Z, Azmi NL, Bull AMJ. Validation and use of a musculoskeletal gait model to study the role of functional electrical stimulation. IEEE Trans Biomed Eng. 2019;66(3):892–7. doi: 10.1109/TBME.2018.2865614 30183617

[23] Long MJ, Papi E, Duffell LD, McGregor AH. Predicting knee osteoarthritis risk in injured populations. Clin Biomech (Bristol). 2017;47:87–95. doi: 10.1016/j.clinbiomech.2017.06.001 28618311 PMC5544598

[24] Duffell LD, Hope N, McGregor AH. Comparison of kinematic and kinetic parameters calculated using a cluster-based model and Vicon’s plug-in gait. Proc Inst Mech Eng H. 2014;228(2):206–10. doi: 10.1177/0954411913518747 24449800

[25] Toderita D, Favier CD, Henson DP, Vardakastani V, Sherman K, Bennett AN, et al. Hip joint and muscle loading for persons with bilateral transfemoral/through-knee amputations: biomechanical differences between full-length articulated and foreshortened non-articulated prostheses. J Neuroeng Rehabil. 2023;20(1):169. doi: 10.1186/s12984-023-01296-4 38115144 PMC10729544

[26] Ding Z, Jarvis HL, Bennett AN, Baker R, Bull AMJ. Higher knee contact forces might underlie increased osteoarthritis rates in high functioning amputees: a pilot study. J Orthop Res. 2021;39(4):850–60. doi: 10.1002/jor.24751 32427347

[27] Bogert AJ, Koning J. On optimal filtering for inverse dynamics analysis. In: Proceedings of the IXth Biennial Conference of the Canadian Society for Biomechanics, Vancouver. 1996.

[28] Yu B, Gabriel D, Noble L, An KN. Estimate of the optimum cutoff frequency for the Butterworth low-pass digital filter. J Appl Biomech. 1999;15:318–29. doi: 10.1123/JAB.15.3.318

[29] Ding Z, Nolte D, Kit Tsang C, Cleather DJ, Kedgley AE, Bull AMJ. In vivo knee contact force prediction using patient-specific musculoskeletal geometry in a segment-based computational model. J Biomech Eng. 2016;138(2):021018. doi: 10.1115/1.4032412 26720641

[30] Cleather DJ, Bull AMJ. The development of a segment-based musculoskeletal model of the lower limb: introducing FreeBody. R Soc Open Sci. 2015;2(6):140449. doi: 10.1098/rsos.140449 26543569 PMC4632533

[31] Ding Z, Tsang CK, Nolte D, Kedgley AE, Bull AMJ. Improving musculoskeletal model scaling using an anatomical atlas: the importance of gender and anthropometric similarity to quantify joint reaction forces. IEEE Trans Biomed Eng. 2019;66(12):3444–56. doi: 10.1109/TBME.2019.2905956 30932815 PMC7100011

[32] Handsfield GG, Meyer CH, Hart JM, Abel MF, Blemker SS. Relationships of 35 lower limb muscles to height and body mass quantified using MRI. J Biomech. 2014;47(3):631–8. doi: 10.1016/j.jbiomech.2013.12.002 24368144

[33] Rajagopal A, Dembia CL, DeMers MS, Delp DD, Hicks JL, Delp SL. Full-body musculoskeletal model for muscle-driven simulation of human gait. IEEE Trans Biomed Eng. 2016;63(10):2068–79. doi: 10.1109/TBME.2016.2586891 27392337 PMC5507211

[34] Azmi NL, Ding Z, Xu R, Bull AMJ. Activation of biceps femoris long head reduces tibiofemoral anterior shear force and tibial internal rotation torque in healthy subjects. PLoS One. 2018;13(1):e0190672. doi: 10.1371/journal.pone.0190672 29304102 PMC5755889

[35] Pataky TC, Robinson MA, Vanrenterghem J. Vector field statistical analysis of kinematic and force trajectories. J Biomech. 2013;46(14):2394–401. doi: 10.1016/j.jbiomech.2013.07.031 23948374

[36] Papi E, Bull AMJ, McGregor AH. Alteration of movement patterns in low back pain assessed by statistical parametric mapping. J Biomech. 2020;100:109597. doi: 10.1016/j.jbiomech.2019.109597 31928738 PMC7001037

[37] Penny W, Friston K, Ashburner J, Kiebel S, Nichols T. Statistical parametric mapping: The analysis of functional brain images. Elsevier Ltd; 2007. doi: 10.1016/B978-0-12-372560-8.X5000-1

[38] Ghédira M, Albertsen IM, Mardale V, Loche C-M, Vinti M, Gracies J-M, et al. Agonist and antagonist activation at the ankle monitored along the swing phase in hemiparetic gait. Clin Biomech (Bristol). 2021;89:105459. doi: 10.1016/j.clinbiomech.2021.105459 34438333

[39] Akl A-R, Hassan A, Elgizawy H, Tilp M. Quantifying coordination between agonist and antagonist elbow muscles during backhand crosscourt shots in adult female squash players. Int J Environ Res Public Health. 2021;18(18):9825. doi: 10.3390/ijerph18189825 34574748 PMC8467896

[40] Souissi H, Zory R, Bredin J, Gerus P. Comparison of methodologies to assess muscle co-contraction during gait. J Biomech. 2017;57:141–5. doi: 10.1016/j.jbiomech.2017.03.029 28433389

[41] Moore D, Semciw AI, Pizzari T. A systematic review and meta-analysis of common therapeutic exercises that generate highest muscle activity in the gluteus medius and gluteus minimus segments. Int J Sports Phys Ther. 2020;15(6):856–81. doi: 10.26603/ijspt20200856 33344003 PMC7727410

[42] Lanza MB, Arbuco B, Ryan AS, Shipper AG, Gray VL, Addison O. Systematic review of the importance of hip muscle strength, activation, and structure in balance and mobility tasks. Arch Phys Med Rehabil. 2022;103(8):1651–62. doi: 10.1016/j.apmr.2021.12.008 34998714 PMC10089299

[43] Brough LG, Neptune RR. Individual muscle responses to mediolateral foot placement perturbations during walking. J Biomech. 2022;141:111201. doi: 10.1016/j.jbiomech.2022.111201 35764014

[44] Davoudi M, Salami F, Reisig R, Gather KS, Wolf SI. Gluteus medius muscle activation patterns during gait with Cerebral Palsy (CP): a hierarchical clustering analysis. PLoS One. 2025;20(1):e0309582. doi: 10.1371/journal.pone.0309582 39787154 PMC11717221

[45] Park SH, Hsu C-J, Dee W, Roth EJ, Rymer WZ, Wu M. Enhanced error facilitates motor learning in weight shift and increases use of the paretic leg during walking at chronic stage after stroke. Exp Brain Res. 2021;239(11):3327–41. doi: 10.1007/s00221-021-06202-9 34477919 PMC8541925

[46] Oh DG, Yoo KT. Effects of functional electrical stimulation (FES) on the temporal-spatial gait parameters and activities of daily living in hemiplegic stroke patients. Korean Society of Physical Medicine. 2021;16:37–44. doi: 10.13066/KSPM.2021.16.3.37

[47] Warshaw ME, Baltz MJ, Hollman JH. Gait synchronized neuromuscular electrical stimulation to the gluteus medius on a patient with right hemiparesis: a case report. Physiother Theory Pract. 2022;38(13):3180–6. doi: 10.1080/09593985.2021.1946874 34260331

[48] Araki S, Kawada M, Miyazaki T, Nakai Y, Takeshita Y, Matsuzawa Y, et al. Effect of functional electrical stimulation of the gluteus medius during gait in patients following a stroke. Biomed Res Int. 2020;2020:8659845. doi: 10.1155/2020/8659845 35721669 PMC9201370

[49] Chung Y, Kim J-H, Cha Y, Hwang S. Therapeutic effect of functional electrical stimulation-triggered gait training corresponding gait cycle for stroke. Gait Posture. 2014;40(3):471–5. doi: 10.1016/j.gaitpost.2014.06.002 24973142

